# Chagas Disease in a Non-Endemic Setting: Clinical Profile, Treatment Outcomes, and Predictors of Cure in a 15-Year Cohort Study

**DOI:** 10.3390/tropicalmed10060161

**Published:** 2025-06-11

**Authors:** Carlos Bea-Serrano, Ana Isabel de Gracia-León, Jara Llenas-García, Sara Vela-Bernal, Andreu Belmonte-Domingo, Carolina Pinto-Pla, Ana Ferrer-Ribera, María José Galindo, María Jesús Alcaraz, María Rosa Oltra Sempere

**Affiliations:** 1 Infectious Disease Unit, Internal Medicine Department, Clinic University Hospital of Valencia, 46010 Valencia, Spain; carlos.bea@outlook.com (C.B.-S.); sara.vela.b@gmail.com (S.V.-B.); anbeldo@hotmail.com (A.B.-D.); cara2384@hotmail.com (C.P.-P.); ferrerllusar@hotmail.com (A.F.-R.); galindo.pepa1@gmail.com (M.J.G.); mrosaoltra@gmail.com (M.R.O.S.); 2 INCLIVA Biomedical Research Institute, 46010 Valencia, Spain; 3 Internal Medicine Department, Vega Baja Hospital, 03314 Orihuela, Spain; 4 Foundation for the Promotion of Health and Biomedical Research of the Valencian Community (FISABIO), 46020 Valencia, Spain; 5 Biomedical Research Networking Center of Infectious Diseases (CIBERINFEC), Carlos III Institute, 28029 Madrid, Spain; 6 Clinical Medicine Department, Miguel Hernández University, 03202 Elche, Spain; 7 Microbiology Service, Clinic University Hospital of Valencia, 46010 Valencia, Spain; mjalcaraz3@gmail.com

**Keywords:** *Trypanosoma cruzi*, Chagas disease, non-endemic cohort, Chagas cardiomyopathy, neglected tropical disease, benznidazole

## Abstract

This retrospective cohort study aimed to assess clinical and epidemiological characteristics, treatment outcomes, and predictors of serological cure in patients with chronic Chagas disease in a non-endemic setting. All individuals aged ≥16 years with confirmed infection and evaluated at a tertiary hospital in Spain from 2008 to 2023 were included. Most of the 107 participants were women (78.5%) and Bolivian-born (99.1%). Digestive and cardiac involvement were identified in 32.7% and 17.8% of cases, respectively. Cardiac symptoms were significantly associated with the diagnostic findings of cardiac involvement (odds ratio [OR] 3.0, 95% confidence interval [CI] 1.1–8.2), whereas digestive symptoms did not correlate with imaging abnormalities (OR 0.7, 95% CI 0.3–1.6). Antiparasitic treatment, usually benznidazole, was initiated in 69% of patients and led to adverse events in 66.2%, with treatment discontinuation in 25.7%. Only 8.1% of treated patients achieved serological cure after a median 26 months, with obesity emerging as the only independent predictor (adjusted OR 31.0, 95% CI 3.7–261.2). Cardiac progression occurred in 9.3% of patients despite treatment. Although 59.8% were lost to follow-up, the cohort maintained a median follow-up of 27 months. These findings underscore the need for improved treatment strategies and sustained clinical monitoring in non-endemic settings.

## 1. Introduction

American trypanosomiasis, or Chagas disease (CD), is a potentially fatal condition caused by the protozoan parasite *Trypanosoma cruzi* naturally transmitted by triatomine vectors in endemic countries [1]. Despite advances in vector control, an estimated 6–7 million people remain infected globally, with approximately 10,000 deaths annually due to Chagas cardiomyopathy [2]. Originally confined to rural Latin America, the disease urbanized in the mid-20th century and subsequently spread worldwide through migration and globalization [3]. Today, CD represents an emerging public health concern in non-endemic regions, including the United States and Europe. Moreover, recent reports have described locally acquired cases in the southern United States, raising concerns about the potential impact of climate change on vector distribution and autochthonous transmission risk [4,5,6]. Spain is the most affected country in Europe, with over 55,000 estimated cases—of which up to 71% may remain undiagnosed [7]. Chronic complications, particularly cardiac and digestive forms, occur in 25–40% of infected individuals in endemic areas [8,9,10,11,12,13,14,15]. In non-endemic settings, however, patients tend to be younger and present with fewer complications. Variability across reports often reflects differences in diagnostic protocols, especially regarding digestive assessment [16,17,18,19,20,21,22].

Although antiparasitic treatment has proven efficacy in acute, congenital, pediatric, and reproductive-age cases [23], its benefit in chronic disease remains controversial [24,25,26,27]. However, several publications [28,29,30,31,32,33,34,35] have related the benefits of etiological treatment for recent chronic infections [31,32,36] and asymptomatic patients [30,31,32,33]. Furthermore, available therapies are limited by frequent adverse events and poor tolerability [22,37,38,39,40]. Reliable early markers of therapeutic success are lacking; serological negativization, the standard indicator of being cured, may take decades [23]. Molecular techniques such as real-time PCR show promise for detecting treatment failure but suffer from limited sensitivity and occasional reversion to positivity over time [16,41,42,43,44]. In addition to its biological impact, CD profoundly affects the emotional and social dimensions of patients’ lives, particularly among marginalized populations, emphasizing the importance of comprehensive long-term management strategies [45]. Given these diagnostic, therapeutic, and monitoring challenges, particularly in non-endemic areas, our study aimed to characterize the clinical and epidemiological features of CD in a Spanish tertiary hospital and identify the factors associated with treatment response, retention during follow-up, and serological cure.

## 2. Materials and Methods

### 2.1. Study Design and Setting

This retrospective observational cohort study took place at a tertiary referral hospital in Valencia, Spain. The hospital provides care for a catchment population of 321,793 people, including 51,197 migrants from Central and South America. All patients aged ≥ 16 years with serologically confirmed CD who were evaluated between June 2008 and February 2023 were included. There was no minimum length of follow-up or pre-determined sample size. The study was approved by the Ethics Committee of INCLIVA Health Research Institute (protocol number 2023/106, 27 March 2023).

### 2.2. Data Collection and Definitions

Clinical, epidemiological, and laboratory data were extracted from electronic medical records. CD was diagnosed using two serological tests: a chemiluminescent immunoassay (LIAISON XL Murex Chagas, DiaSorin, Saluggia, Italy) and an indirect immunofluorescence assay (IFA IgG+IgM, Vircell, Granada, Spain) [46]. *T. cruzi* DNA was assessed using real-time PCR (qPCR) (RealCycler Chagas Kit, Progenie Molecular, Valencia, Spain) [47] at baseline and after treatment. Initial evaluation included blood and serological tests (HIV, HBV, HCV), PCR, electrocardiogram (EKG), chest X-ray, echocardiography, and gastrointestinal imaging with barium enema and esophagogram. Digestive involvement was classified according to Rezende’s stages and colonic grades. Cardiac involvement was assessed using the Kuschnir classification.

### 2.3. Treatment and Outcome

Antiparasitic treatment was offered to all eligible patients, excluding those with severe cardiomyopathy or pregnancy. Benznidazole was administered as first-line therapy (5 mg/kg/day, maximum 300 mg/day for 60 days), with a progressive dose escalation during the first week [48]. Patients with intolerance received reduced doses or alternative regimens: nifurtimox (10 mg/kg/day, max 600 mg/day for 60 days) or, in selected cases, posaconazole (400 mg every 12 h for 60 days as compassionate use). Cure was defined as serological negativization by indirect immunofluorescence assay (IFA).

### 2.4. Statistical Analysis

Categorical variables are presented as frequencies and percentages; continuous variables as means with standard deviation (SD) or medians with interquartile ranges (IQR), as appropriate. The Chi-square or Fisher’s exact tests were used for categorical comparisons; *t*-tests or Mann–Whitney U tests for continuous variables. Kaplan–Meier survival analysis was performed to estimate the probability of retention in clinical follow-up over time. Patients who remained under active follow-up at the time of analysis were administratively censored at their last recorded visit. Survival probabilities at predefined time points (6, 12, 24, and 60 months) were estimated using the life-table method with user-defined intervals of six months. Logistic regression was used to identify factors associated with serological cure. Statistical significance was set at *p* < 0.05. All statistical analyses, including survival curve and life-table generation, were performed using SPSS version 30.0.

## 3. Results

We included 107 patients with chronic CD. Most were women (78.5%) with a median age of 38 years (IQR 33–46) and 99.1% were born in Bolivia. Table 1 summarizes their epidemiological characteristics. Among women, 95.2% had children, and 50% of these children were tested for CD, with a 5% positivity rate. The main comorbidities and analytical findings are summarized in Table 1. Serological testing was performed in 82.2% of patients for HBV, 80.4% for HCV, and 25.2% for *Strongyloides*, with resolved HBV infection found in 11.2% and *Strongyloides* infection in 11.2%. HIV serology was performed in all patients, with one positive case (viral load 424,000 copies, CD4 count 60 cells/mL). There were two other immunocompromised patients (one with non-Hodgkin lymphoma and one with systemic lupus erythematosus).

Cardiac symptoms were reported in 31.8% of patients at the initial evaluation and digestive symptoms in 34.6% (Table 2). Ninety-five patients (88.8%) underwent chest X-rays, with 81.3% showing normal results, while 4.7% had cardiomegaly and 1.1% exhibited other abnormalities. EKGs were performed in 99 patients (92.5%), among whom 81.8% showed normal results. The most frequent abnormalities included rsR’ patterns (a secondary R wave in lead V1 with normal QRS duration, suggestive of incomplete right bundle branch block) (8.4%), right bundle branch block (6.5%), and left anterior fascicular block (1.9%). Transthoracic echocardiography was conducted in 85 patients (79.4%), with normal findings in 64.7% of these. Common alterations included mild tricuspid insufficiency (9.3%), mild mitral insufficiency (5.6%), and mild pulmonary hypertension (2.8%). Additional findings, such as pericardial effusion and ventricular or atrial dilation, were rare (≤2.8%). Only one patient underwent cardiac MRI, which revealed atrial and ventricular dilation (0.9%). However, only 19 patients (17.8%) were classified as having Chagas cardiomyopathy at diagnosis based on Kuschnir classification (Table 2). Among the 19 patients with initial cardiac involvement based on EKG, chest X-ray, or transthoracic echocardiography (TTE) abnormalities, 52.6% had cardiac symptoms. Conversely, 12.3% of patients without cardiac symptoms showed cardiac involvement in complementary studies. The cross-tabulation analysis revealed a significant association between cardiac symptoms and evidence of cardiac involvement in complementary studies (odds ratio [OR] 3.0, 95% confidence interval [CI] 1.1–8.2; *p* = 0.031).

Treatment was initiated in 74 patients (69.2%), mainly with benznidazole (94.6%). Twenty-two patients (29.7%) discontinued treatment, mostly due to adverse events. These were reported in 66.2% (49/74) of patients receiving first-line treatment. The most frequent side effect was exanthema (32.4%), followed by nausea and headache (14.9% each), epigastric pain (10.8%), pruritus (8.1%), general malaise (6.8%), and arthromyalgias (6.8%). Less commonly reported events included fever (5.4%), paresthesia (2.7%), and isolated cases of diarrhea, lip edema, skin desquamation, asthenia, and oral ulcers (1.4% each or less). These adverse effects led to treatment discontinuation in 19 patients (25.7%), while 3 more discontinued for unrelated reasons. Thirty patients (28%) received second-line treatment with nifurtimox, mainly due to rising or persistently elevated titers (70%, 21/30) and benznidazole intolerance (23.3%, 7/30). Adverse events occurred in 22 of these patients (73.3%), leading to nifurtimox discontinuation in 33.3% (10/30). Regarding the compassionate use of posaconazole, 17 patients (15.9%) received it as a third-line treatment, mainly due to rising or persistently elevated titers (41.2%, 7/17) and second-line treatment intolerance (41.2%, 7/17). It showed a better tolerance profile with a lower rate of adverse events (47.1%, 8/17) and fewer discontinuations (11.8%, 2/17).

**Figure 1:**
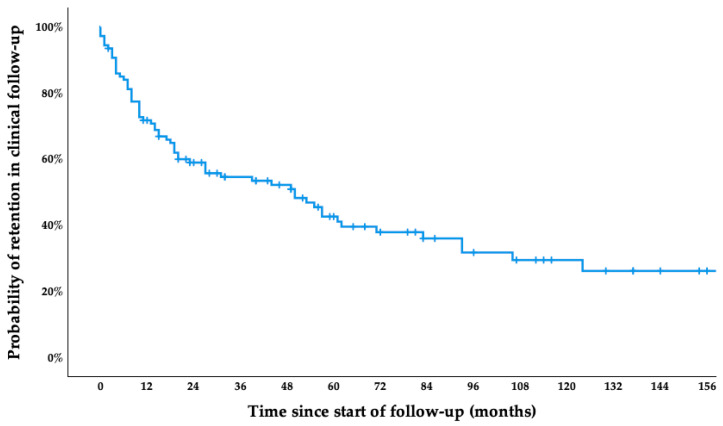
Kaplan–Meier survival curve showing retention in clinical follow-up among patients with chronic CD after diagnosis.

During the study period, 59.8% of patients were lost to follow-up, while the rest remained under active clinical monitoring at the time of data analysis. The reasons for loss to follow-up were mainly unknown (49 patients, 76.6%), while some returned to their home country (5 patients, 7.8%), transferred to another autonomous community within Spain (4 patients, 6.3%), transferred to another hospital (3 patients, 4.7%), or died (2 patients, 3.1%)—one due to severe COVID-19 pneumonia and the other from an unknown cause. The median follow-up time was 27 months (IQR 10–62). The cumulative probability of remaining under clinical monitoring was approximately 72% at 12 months, 54% at 24 months, 43% at 48 months, and 38% at 60 months. By 180 months, the estimated probability of retention had declined to 26% (Figure 1). Differences in follow-up retention according to baseline clinical characteristics—including sex, age, the presence of cardiac or digestive symptoms, evidence of cardiac or digestive involvement on complementary examinations, and the presence of comorbidities—were assessed using the log-rank test. No statistically significant differences were observed. The complete log-rank analysis is available in Supplementary Material File S1.

Post-treatment qPCR for *T. cruzi* was performed in 40.5% of treated patients, with 93.4% of initially qPCR-positive patients achieving negativization at six months after treatment. Serological negativization was achieved in 6 of the 74 treated patients (8.1%) and took a median time of 26 months (IQR 4–37). Of note, the median length of follow-up among patients who achieved serological cure was 67 months (IQR 17.5–126), longer than that of the overall cohort. Notably, none of these cured patients had received treatment prior to arriving at our center. Table 3 presents the analysis of factors associated with serological negativization. In the multivariate analysis, obesity was the only factor independently associated with cure (adjusted OR 31.0, 95% CI 3.7–261.2; *p* = 0.002). Disease progression occurred in 10 patients (9.3%) during follow-up, over a median time of 82.5 months (IQR 53–121), and all had received at least one line of treatment. Progression involved cardiac complications such as three cases of right-bundle branch block, one case of atrioventricular block, one case of sinus dysfunction requiring pacemaker implantation, and one case of ventricular fibrillation necessitating ICU admission and the implantation of a dual-chamber implantable cardioverter defibrillator. Of these progressing patients, six had received three treatment lines, and four had been treated with only one. None of the three immunocompromised patients experienced reactivation during follow-up.

## 4. Discussion

This study provides a comprehensive characterization of CD in a non-endemic European setting, with systematic clinical assessment and follow-up over 15 years, one of the longest follow-ups reported outside endemic areas. Two-thirds of treated patients had drug-related adverse events, leading to discontinuation in a quarter of them. We found a low rate of serological cure over the follow-up period, and almost 10% of patients experienced cardiac progression despite having received antiparasitic treatment. Patient retention in clinical follow-up progressively declined over time, despite systematic efforts to maintain engagement.

The clinical heterogeneity of CD patients described in the literature across endemic and non-endemic areas, as well as among different non-endemic regions, has been attributed to varying management protocols and the geographical distribution of the endemic populations residing in non-endemic areas [49]. Our population is comparable to the largest non-endemic series published to date, conducted in Barcelona by Salvador et al. and Laynez-Roldan et al. [22,50], with similar distributions of sex, age, and predominant country of origin (over 85% Bolivian).

Cardiac involvement in our study aligns with the majority of non-endemic series (16–20%) [18,20,22,50]. Fewer patients in Salvador et al.’s [22] series reported cardiac symptoms (13.8% vs. 31.8%). However, evidence of cardiac involvement on chest X-rays or EKGs was comparable to that in the cohorts from Barcelona (16.8% vs. 16.9% in Salvador et al. [22] and 17.1% in Laynez-Roldan et al. [50]), and the distributions of Kuschnir classification groups were nearly identical. Additionally, the percentage of patients who underwent echocardiography is not reported by Salvador et al. [22], whereas Laynez-Roldan et al. [50] reported that it was performed in only 12.9% of patients with CD. The most frequent EKG alteration in our cohort, as well as in Laynez-Roldan et al.’s [50], was the right bundle branch block, whereas in other series, such as Salvador et al.’s [22], it was sinus bradycardia. In contrast, recent data from the United States suggest a different clinical profile: in a cohort from New York City, EKG and echocardiogram abnormalities were observed in 57% and 54% of patients, respectively [51], while a multicenter study reported cardiac involvement in 66.6% of cases [52]. These figures are markedly higher and approach those historically reported in endemic regions [15]. These differences could be partly explained by the “healthy migrant effect” [16,18], by the different age of the patients in the different cohorts, by social determinants and environmental factors that could contribute to CD progression and by clinical variations between countries with different *T. cruzi* DTU profiles [17,19,53]. Consistent with the main international guidelines [54,55,56], chest X-rays and EKGs, which remain key, accessible tools for the early detection of subclinical cardiac damage—even in asymptomatic patients [57], should be routinely performed at diagnosis and during follow-up due to the severity of cardiac involvement. Given the progressive nature of Chagas cardiomyopathy, even in initially asymptomatic patients, long-term clinical monitoring remains essential. Recent estimates indicate an annual risk of progression from the indeterminate form to cardiomyopathy of approximately 1.9% [58], underscoring the importance of sustained surveillance beyond initial diagnosis and treatment [57]. In our series, 10 patients (9.3%) experienced cardiac progression despite having received antiparasitic treatment, with two requiring pacemaker implantations, one of whom also presented with severely reduced LVEF. Notably, most of these patients (60%) had received three lines of treatment due to rising or persistently elevated serological titers, suggesting a potential association between serological non-response and cardiac progression, as well as the futility of retreating patients with additional lines after a treatment failure.

Digestive involvement rates in endemic regions vary widely (10–34%) [8,9,10,15], but they are generally lower in non-endemic areas (1–10%) [20,21,59]. However, the proportion of patients with digestive involvement was higher in our study (32.7%), as was the proportion with dolichocolon (24.3%) or megacolon (8.4%) identified through imaging, probably due to the systematic use of barium enema and esophagogram for evaluating digestive involvement in all patients, the high percentage of female patients [60], and the possibly greater prevalence of discrete typing units (DTUs) TcII [61,62], TcV, and TcVI [62,63], as TcV has been reported as the most frequent DTU among Bolivian patients with Chagas disease living in Spain [64,65,66]. Conversely, the percentage of patients with normal esophagograms was nearly identical in our series compared with those with available data (95–97%). Consistent with the findings of previous studies, ours suggests that esophagograms are unlikely to be cost-effective in asymptomatic patients. Likewise, the high proportion of dolichocolon cases without a significant clinical impact in our cohort suggests that barium enema may not be universally justified. Table 4 shows the comparison between the different series published in non-endemic areas.

Although Chagas reactivation in immunocompromised patients with severe clinical manifestations has been increasingly reported [68,69,70], none of the three immunocompromised patients in our series exhibited reactivation during follow-up. Co-infection with *Strongyloides stercoralis* is increasingly recognized among individuals with chronic Chagas disease. A large retrospective study in Spain found that *T. cruzi* infection was independently associated with a higher risk of *Strongyloides* infection (OR 2.23, 95% CI 1.07–4.64) [71]. Despite this well-established relationship, only 25.2% of our patients underwent *Strongyloides* serology. Still, 11.2% of the entire cohort tested positive, representing 44.4% of those screened. This underscores the need to incorporate systematic *Strongyloides* screening into the clinical management of Chagas disease, especially given the risks of hyperinfection in immunosuppressed people.

Current treatment recommendations for CD in adults remain limited and are based on a few clinical trials. Although antiparasitic treatment with benznidazole may modestly reduce disease progression in early stages of CD and indeterminate clinical forms [34,35]—with some studies from endemic areas showing benefit even in patients with mild cardiomyopathy but no heart failure [29,72]—the randomized controlled trial BENEFIT did not show a significant benefit in patients with established cardiomyopathy [57]. At our center, treatment is proactively offered to all patients except those with severe established Chagas cardiomyopathy or pregnant women. As a result, 69% of patients received at least one line of treatment, which is higher than in Salvador et al. (51.1%) and most of the other published series [18,20,21], only surpassed by the Italian series from Milano in Gobbi et al. [67], which treated 96.7% of the patients. In contrast, recent North American cohorts reported lower treatment rates: 45% in the New York City cohort described by Zheng et al. [51] and only 3% in the recent multicenter study by Henao-Martínez et al. [52], although, in the latter, treatment initiation was evaluated only within the first six months after serological diagnosis. Adverse effects from benznidazole occurred in 66.2% of cases, higher than rates reported in endemic countries for Chagas disease [27,32,73,74] and other non-endemic (27.7–57%) [37,38,39,40,67], but similar to those reported by Salvador et al. (72.6%) [22], although treatment discontinuation due to toxicity was greater in our series (25.7% vs. 13.7%). Nifurtimox, mainly used as a second-line treatment, was slightly worse tolerated, with 73.3% patients experiencing adverse events and 33.3% discontinuing treatment—findings in a similar range to those reported in other studies [75,76]. A small group of patients in our cohort received posaconazole on a compassionate-use basis between 2008 and 2014, prior to the randomized trial by Molina et al. that highlighted its limited efficacy [77]. At that time, its off-label use in refractory cases or when benznidazole or nifurtimox had to be discontinued due to intolerance was supported by evidence of trypanocidal effects in murine models [78] and scarce clinical reports [79]. Given the substantial limitations in the tolerability of current antiparasitic regimens [37,38,75,77,80], recent therapeutic developments have sought to identify safer alternatives [81]. However, the FEXI-12 phase 2 trial, one of the most recent and rigorously conducted studies evaluating fexinidazole monotherapy for chronic CD, failed to demonstrate efficacy [82].

Despite the large proportion of patients in our series who received treatment, this led to serological negativization (cure) in just 8.1%. No clear trend of declining serological titers was observed in most treated patients, even 5–6 years post-treatment, with some patients showing increases in previously stable titers. For patients in whom serological negativization occurred, the median time to cure was 26 months (IQR 4–37), data that were not provided for other non-endemic cohorts [18,20,21,22,50]. In a systematic review of follow-up in patients treated for CD, Sguassero et al. [26] reported that ELISA test negativization showed highly variable data, particularly between 12 and 36 months after treatment, but the curve increased to 5% serological negativization at 12 months, 10% at 24 months, and approximately 15% at 48 months, reaching almost 45% in the long-term follow-up (264 months). Notably, that study included children, who have shown a higher cure rate and the earlier negativization of serological tests than adults. In a recent observational comparative study in an endemic area with 1497 adults diagnosed with chronic CD over a 14-year period (1967–1980), nifurtimox-treated patients demonstrated a shorter median time to serological negativization (2.1 years) than untreated patients (2.4 years) [76]. Evidence from a recent meta-analysis indicates that serological negativization in adults treated during the chronic phase of Chagas disease usually take decades, with particularly prolonged times reported in Brazilian series [83]. Given the prolonged time to serological negativization observed after treatment, one of the major challenges in the management of CD remains the absence of reliable early markers of cure. Although PCR-based assays have been explored as surrogate markers, their sensitivity is limited by the intermittent and low-level parasitemia characteristic of chronic infection, leading to a substantial risk of false negatives [41,43,84] and indicating only a substantial decrease in parasitemia [85,86]. Additionally, in our cohort, post-treatment qPCR was performed on only five patients who received nifurtimox and one patient who received posaconazole, thus preventing any meaningful comparison of negativization rates across the different treatments. Consequently, the need for novel, more accurate biomarkers of therapeutic response is critical to optimizing patient management and facilitating clinical trials [87]. Addressing this gap, initiatives like the recently launched ChaNoE cohort in Spain, which systematically collects clinical data and biobank samples to identify biomarkers of cure and disease progression [88], and the TESEO trial in Bolivia, which compares treatment regimens and explores biomarker discovery [89], represent promising steps forward.

In our study, obesity was significantly associated with serological cure in both bivariate and multivariate analyses. Although this finding may seem counterintuitive—especially considering that our center caps the maximum daily benznidazole dose at 300 mg regardless of weight—it could hypothetically be explained by pharmacokinetic factors, such as the lipophilic properties of benznidazole favoring drug accumulation in adipose tissue. However, baseline serological titers were similar between obese and non-obese patients, and no significant differences were observed in treatment duration, adverse event rates, or discontinuation rates across these groups. Multivariate logistic regression, performed using stepwise forward and backward selection followed by robust modeling, consistently identified obesity as an independent predictor of cure. To our knowledge, this association has not been previously described in the literature; however, data on weight or BMI are lacking in other cohorts. Recent phase II trials BENDITA [90] and MULTIBENZ [91] did not report BMI differences among their treatment groups. Nonetheless, given the limited number of seronegative conversions and the possibility of residual confounding, this association should be interpreted with caution and warrants confirmation in larger, prospective studies.

Our study has several strengths. It is one of the largest single-center cohorts of Chagas patients in Europe, with systematic diagnostic and therapeutic protocols and long-term follow-up. The availability of detailed clinical data allowed for the exploration of associations with treatment response and disease progression. In addition, this study highlights the need to enhance all measures of Chagas disease transmission control among the European population, encompassing all mechanisms independent of the triatomine vectors. However, we acknowledge some limitations. The retrospective design entails the possibility of incomplete data. Loss to follow-up was considerable, reflecting the mobility of the migrant population and the challenges of long-term retention in care. PCR testing was not consistently performed in all patients, which limits the interpretation of parasite persistence. Finally, the small number of patients achieving cure limited the statistical power of the predictive model.

Our study characterizes chronic CD in a non-endemic setting, highlighting the challenges in management. While cardiac screening was crucial, routine gastrointestinal radiography in asymptomatic patients yielded limited clinical utility. Despite proactive treatment, high rates of adverse events, frequent discontinuations, and rare serological cure were observed, compounded by patient retention issues. The observed association between obesity and serological negativization warrants further investigation for potential individualized therapeutic strategies. These findings underscore the critical need for more effective therapies, reliable biomarkers, and sustained long-term monitoring. Altogether, our results advocate for sustained investment in therapeutic innovation and optimized care models tailored for non-endemic regions.

## Figures and Tables

**Table 1:** Baseline epidemiological and microbiological characteristics of 107 patients with chronic CD followed in a tertiary hospital in Spain.

		n (%) or Median (IQR)
Epidemiological characteristics		
Female sex		84 (78.5%)
Age, median (IQR), years		38.0 (33.0–46.0)
Country of origin	Bolivia	106 (99.1%)
	Venezuela	1 (0.9%)
Area of residence	Rural	65 (60.7%)
	Urban	22 (20.6%)
	Unknown	20 (18.7%)
Transmission route	Unknown	105 (98.1%)
	Blood transfusion	1 (0.9%)
	Vectorial	1 (0.9%)
Family history of CD		62 (57.9%)
Women with offspring (n = 84)	Yes	80 (95.2%)
	Children tested for CD	40 (50.0%)
	Children diagnosed with CD	4 (5.0%)
Clinical characteristics		
Comorbidities	Hypertension	4 (3.7%)
	Diabetes mellitus	3 (2.8%)
	Dyslipidemia	38 (35.5%)
	Obesity (BMI ≥ 30)	6 (5.6%)
	Elevated AST/ALT	8 (7.5%)
	Hyperbilirubinemia	3 (2.8%)
	Hypothyroidism	2 (1.9%)
	Eosinophilia	7 (6.5%)
	Anemia	3 (2.8%)
	Thrombocytopenia	1 (0.9%)
	Iron deficiency	2 (1.9%)
Coinfections	HIV	1 (0.9%)
	HBV (resolved infection)	12 (11.2%)
	HCV	0 (0.0%)
	Strongyloides infection	12 (11.2%)
Immunocompromised status	Hematological malignancy	1 (0.9%)
	SLE with immunosuppressive therapy	1 (0.9%)
	AIDS	1 (0.9%)
Microbiological diagnosis		
Baseline anti-*T. cruzi* antibody titer (IFA)	1:32–1:64	25 (23.4%)
	1:128–1:256	37 (34.5%)
	≥1:512	41 (38.3%)
	Unknown	4 (3.7%)
Baseline *T. cruzi* qPCR	Positive	20 (18.7%)
	Negative	66 (61.7%)
	Not performed	21 (19.6%)

AIDS, acquired immunodeficiency syndrome; ALT, alanine aminotransferase; AST, aspartate aminotransferase; BMI, body mass index; HBV, hepatitis B virus; HCV, hepatitis C virus; IFA, immunofluorescence assay; IQR, interquartile range; qPCR, real-time polymerase chain reaction; SLE, systemic lupus erythematosus.

**Table 2:** Clinical profile at initial consultation of 107 patients with chronic CD followed in a tertiary hospital in Spain.

		n (%)
Cardiac involvement		
Symptoms	Any cardiac symptom	34 (31.8%)
	Palpitations	19 (17.8%)
	Atypical chest pain	16 (15.0%)
	Dyspnea on exertion	10 (9.4%)
	Syncope	2 (1.8%)
	Orthopnea	1 (0.9%)
Kuschnir classification	Group 0: normal EKG and chest X-ray	81 (75.7%)
	Group I: abnormal EKG, normal chest X-ray	17 (15.9%)
	Group II: LV dilation	1 (0.9%)
	Group III: congestive heart failure	1 (0.9%)
	Not classified *	7 (6.5%)
Digestive involvement		
Symptoms	Any digestive symptom	37 (34.6%)
	Heartburn	15 (14.0%)
	Acid reflux	14 (13.1%)
	Flatulence	13 (12.2%)
	Constipation	13 (12.2%)
	Dyspepsia	7 (6.5%)
	Abdominal distension	3 (2.8%)
	Early satiety	2 (1.9%)
Esophageal involvement **	No esophageal involvement	93 (97.9%)
	Rezende I	0
	Rezende II	0
	Rezende III	1 (1.1%)
	Rezende IV	0
Colonic involvement **	No colonic involvement	60 (63.2%)
	Group 1: Dolichocolon	26 (27.4%)
	Group 2: Dolichomegacolon or megacolon	9 (9.5%)

EKG, electrocardiogram; LV, left ventricular; IQR, interquartile range. * Not classified: patients without available EKG. ** Digestive evaluation performed in 95 patients (88.8%) by esophagogram and barium enema.

**Table 3:** The univariate and multivariate analysis of factors associated with cure in CD patients followed in a tertiary hospital in Spain.

Variable		Crude OR (95% CI)	*p*-Value	Adjusted OR (95% CI)	*p*-Value
Age (years)	18–35 (ref.)	1.00	—	1.00	—
	36–45	3.68 (0.39–34.57)	0.254	2.33 (0.16–33.01)	0.532
	≥46	1.25 (0.08–20.89)	0.877	0.33 (0.006–18.91)	0.588
Sex	Female (ref.)	1.00	—	1.00	—
	Male	1.90 (0.33–11.12)	0.474	1.33 (0.10–18.24)	0.832
Hypertension		NC	NC	NC	NC
Diabetes		NC	NC	NC	NC
Dyslipidemia		0.76 (0.08–6.88)	0.762	1.98 (0.14–27.17)	0.610
Obesity		32.67 (4.56–234.22)	0.001	31.00 (3.68–261.28)	0.002
HIV		NC	NC	NC	NC
HBV infection		2.06 (0.20–21.63)	0.547	5.71 (0.27–121.11)	0.263
Initial *T. cruzi* qPCR	Positive (ref.)	0.00	0.998	0.00	0.998
	Negative	1.64 (0.18–14.88)	0.660	2.34 (0.11–49.96)	0.586
1st-line treatment duration	≤60 days (ref.)	1.00	—	1.00	—
	>60 days	5.00 (0.76–32.77)	0.069	3.65 (0.42–32.00)	0.242

OR, odds ratio; CI, confidence interval; NC, not calculable; HBV, hepatitis B virus; HIV, human immunodeficiency virus; qPCR, real-time polymerase chain reaction.

**Table 4:** Comparison of the different series of patients with CD in non-endemic areas.

	Country	Country of Origin	Period	Patients (n)	Age (yrs)	Cardiac Evaluation	Cardiac Abnormalities	Digestive Evaluation	Digestive Abnormalities	Patients Treated
Jackson [20]	Switzerland	Bolivia 93.4%	1979–2011	258	41	-	20.1%	-	0.7%	129 (50%)
Ramos [21]	Spain	Bolivia 78.9%	2002–2011	128	35	EKG, CXR, TTE	24.1%	BE, esophagogram, if symptoms	0.9%	76 (59.3%)
Laynez-Roldan [50]	Spain	Bolivia 86,6%	2002–2019	1382	36.8	EKG, TTE	17.1%	BE, esophagogram, if symptoms	2.6%	-
Pérez-Ayala [18]	Spain	Bolivia 97%	2003–2009	357	36	EKG, TTE	18.6%	BE, esophagogram, if symptoms	5.1%	195 (54.6%)
Muñoz [16]	Spain	Bolivia 86.6%	2004–2007	202	36	EKG, CXR	19%	BE, esophagogram, if symptoms	9%	-
Valerio [19]	Spain	Bolivia 95%	2005–2009	100	38.2	EKG, TTE	49%	-	-	-
Zheng [34]	United States	El Salvador 40%, Mexico 20%, Ecuador 12%, Bolivia 12%	2005–2017	60	47	EKG, CXR, TTE	54%	Colonoscopy, abdominal US, if symptoms	6.70%	27 (45%)
Gobbi [67]	Italy	Bolivia 97.3%	2005–2013	332	41.8	EKG, CXR, TTE	11%	BE, esophagogram, all	19%	321 (96.7%)
Salvador [22]	Spain	Bolivia 97%	2007–2012	1274	37.7	EKG, CXR	16.9%	BE, esophagogram, all	14.8%	636 (51.1%)
Lescure [17]	France	Bolivia 87.4%	2008–2009	60	33	Symptoms	23.6%	Symptoms	22%	-
Henao-Martínez [52]	United States	-	2017–2023	429	49.5	EKG, CXR, TTE	66.6%	-	-	3% *
Bea-Serrano	Spain	Bolivia 99.1%	2008–2023	107	39.8	EKG, CXR, TTE	16.8%	BE, esophagogram, all	32.7%	74 (69.2%)

BE, barium enema; CXR, chest X-ray; HEM, hematologic malignancy; HIV, human immunodeficiency virus; ONC, oncologic malignancy; SLE, systemic lupus erythematosus; SOT, solid organ transplantation; TTE, transthoracic echocardiography. * Only treatments initiated within 6 months after a positive *T. cruzi* serology were considered.

## Data Availability

The original contributions presented in this study are included in the article/Supplementary Material. Further inquiries can be directed to the corresponding author.

## Supplementary Materials

The following supporting information can be downloaded at https://www.mdpi.com/article/10.3390/tropicalmed10060161/s1, File S1: Log-rank test analysis for differences in follow-up retention according to baseline clinical characteristics.

## Abbreviations

The following abbreviations are used in this manuscript:

AIDS	Acquired immunodeficiency syndrome
ALT	Alanine aminotransferase
AST	Aspartate aminotransferase
BE	Barium enema
BMI	Body mass index
CD	Chagas disease
CI	Confidence interval
DTU	Discrete typing unit
EKG	Electrocardiogram
ELISA	Enzyme-linked immunosorbent assay
GDPR	General Data Protection Regulation
HBV	Hepatitis B virus
HCV	Hepatitis C virus
HIV	Human immunodeficiency virus
IFA	Indirect immunofluorescence assay
IQR	Interquartile range
ISCIII	Instituto de Salud Carlos III
MRI	Magnetic resonance imaging
OR	Odds ratio
PCR	Polymerase chain reaction
qPCR	Real-time polymerase chain reaction
SD	Standard deviation
SLE	Systemic lupus erythematosus
TTE	Transthoracic echocardiography
