# Supplementary material for: Chagas Disease in a Non-Endemic Setting: Clinical Profile, Treatment Outcomes, and Predictors of Cure in a 15-Year Cohort Study
Source: Trop Med Infect Dis. 2025 Jun 11;10(6):161. doi: 10.3390/tropicalmed10060161 (PMC12197416; doi:10.3390/tropicalmed10060161)

Supplementary material

S1 – Log-Rank test analysis for differences in follow-up retention according to baseline clinical characteristics

Sex

Means and medians for survival

	Mean[a]			Median		
	Estimate	Std. Error	(95% CI)	Estimate	Std. Error	(95% CI)
Female	80.401	8.857	(63.042, 97.760)	57.000	6.594	(44.076, 69.924)
Male	31.930	6.762	(18.676, 45.183)	19.000	3.497	(12.145, 25.855)
Overall	74.173	7.931	(58.628, 89.719)	50.000	11.725	(27.018, 72.982)

a. Estimation is limited to the largest survival time if it is censored.

Overall Comparisons

	Chi-Square	df	P value
Log rank (Mantel-Cox)	3.486	1	0.062

Test of equality of survival distributions for the different levels of SEX.

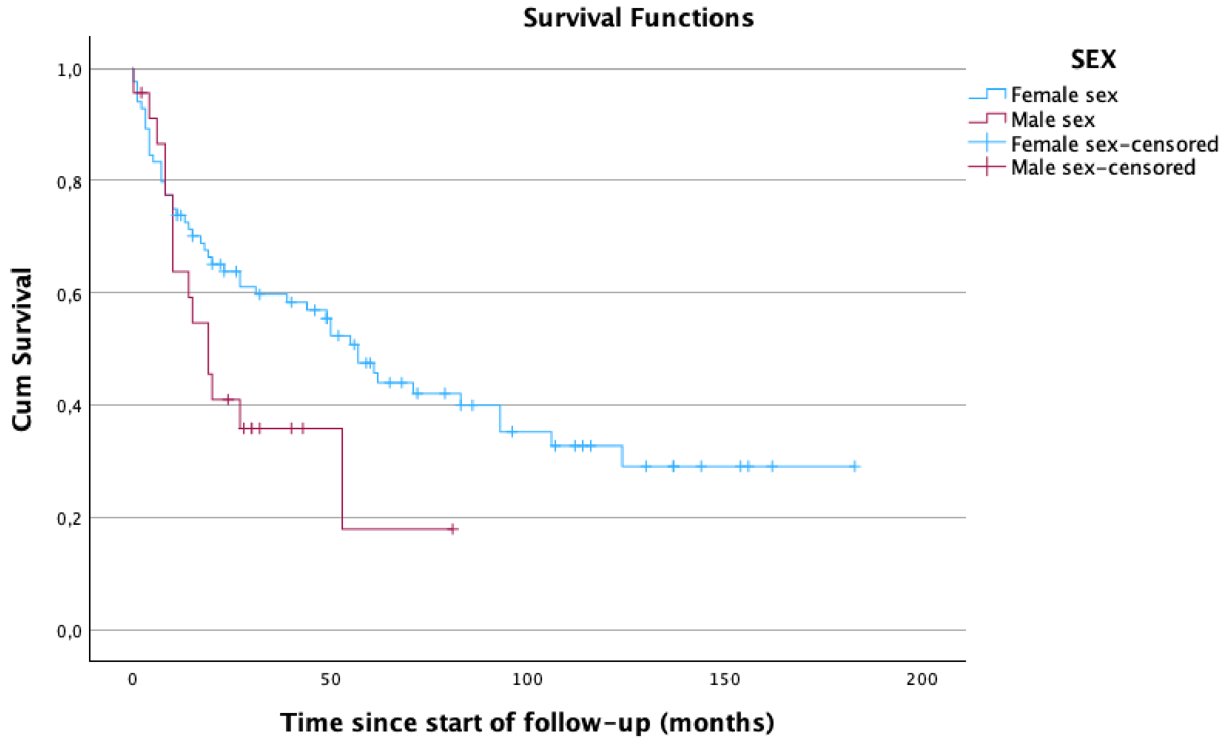

Means and medians for survival, according to age group

	Mean[a]			Median		
AGE GROUP	Estimate	Std. Error	(95% CI)	Estimate	Std. Error	(95% CI)
18-35	67.195	12.753	(42.200, 92.191)	27.000	15.000	(0.000, 56.400)
36-45	59.038	9.574	(40.272, 77.803)	50.000	21.196	(8.455, 91.545)
46 or more	86.782	12.477	(62.327, 111.236)	—	—	—
Overall	74.173	7.931	(58.628, 89.719)	50.000	11.725	(27.018, 72.982)

a. Estimation is limited to the largest survival time if it is censored.

Overall Comparisons

	Chi-Square	df	P value
Log Rank (Mantel-Cox)	3.068	2	0.216

Test of equality of survival distributions for the different levels of AGE GROUP.

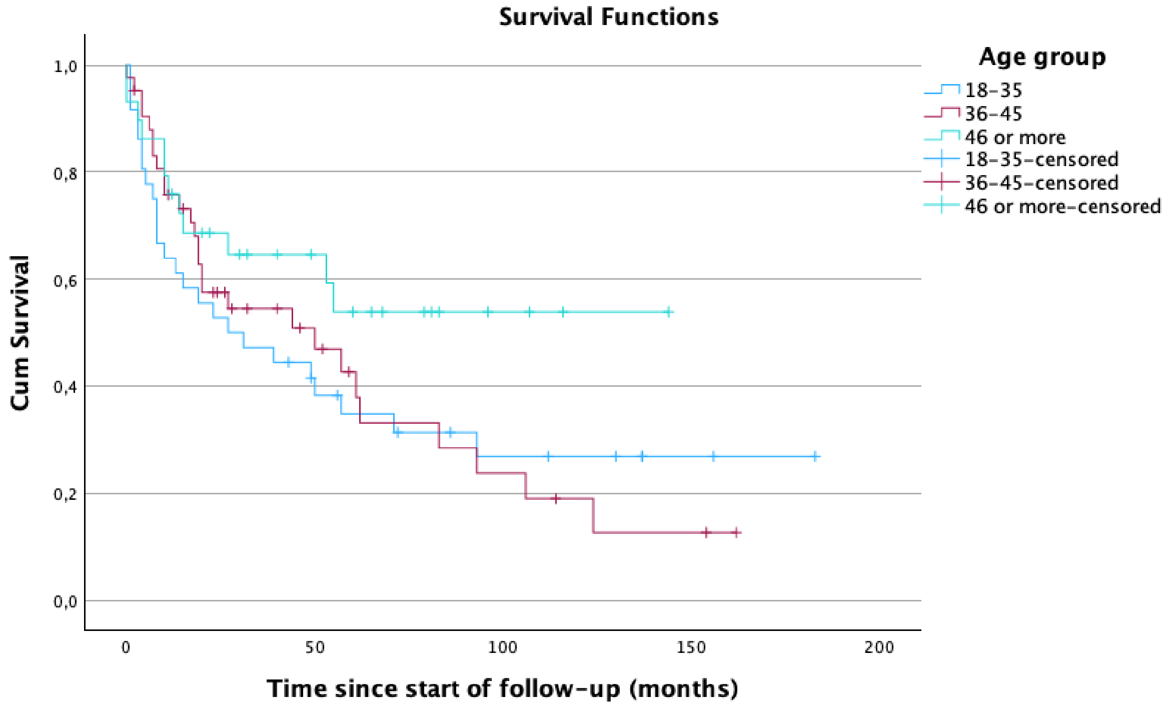

Initial cardiac involvement

Means and medians for survival

	Mean[a]			Median		
Initial cardiac involvement	Estimate	Std. Error	(95% CI)	Estimate	Std. Error	(95% CI)
No	70.376	8.502	(53.712, 87.040)	44.000	12.012	(20.456, 67.544)
Yes	85.620	16.439	(53.400, 117.841)	106.000	30.338	(46.537, 165.463)
Overall	74.173	7.931	(58.628, 89.719)	50.000	11.725	(27.018, 72.982)

a. Estimation is limited to the largest survival time if it is censored.

Overall Comparisons

	Chi-Square	df	P value
Log Rank (Mantel-Cox)	1.440	1	0.230

Test of equality of survival distributions for the different levels of Initial cardiac involvement.

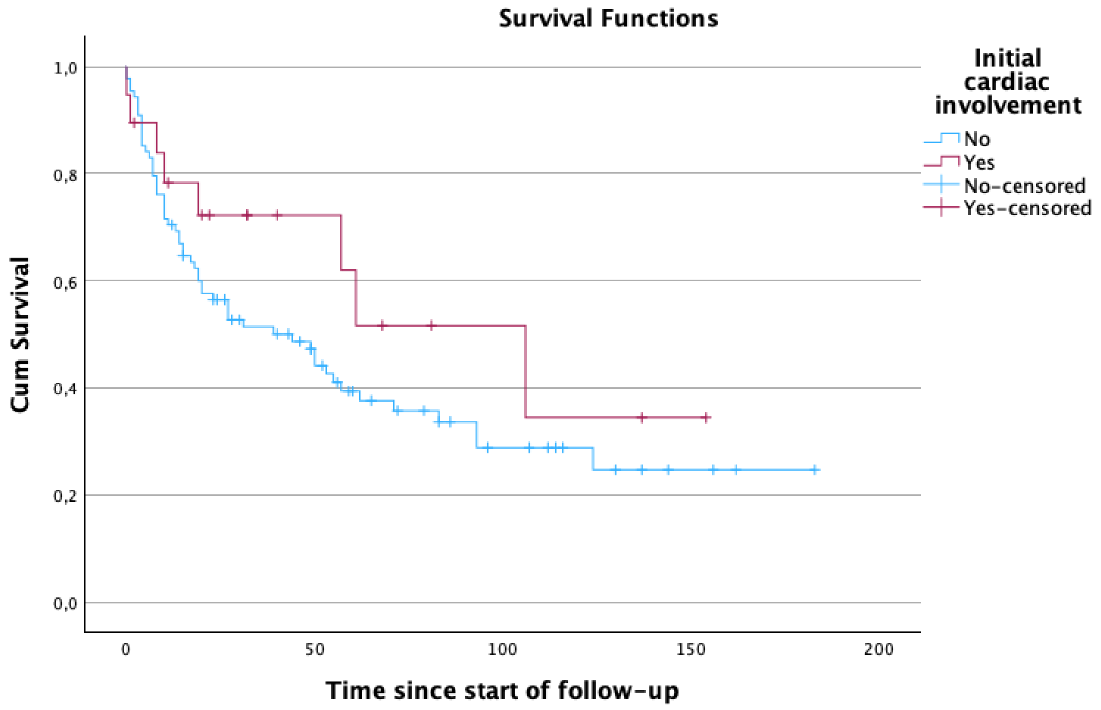

Initial digestive involvement

Means and medians for survival

Initial digestive involvement	Mean[a]			Median		
	Estimate	Std. Error	(95% CI)	Estimate	Std. Error	(95% CI)
No	69.035	9.901	(49.629, 88.442)	31.000	12.756	(5.999, 56.001)
Yes	78.260	11.051	(56.599, 99.921)	83.000	26.079	(31.886, 134.114)
Overall	74.173	7.931	(58.628, 89.719)	50.000	11.725	(27.018, 72.982)

a. Estimation is limited to the largest survival time if it is censored.

Overall Comparisons

	Chi-Square	df	P value
Log Rank (Mantel-Cox)	1.467	1	0.226

Test of equality of survival distributions for the different levels of Initial digestive involvement.

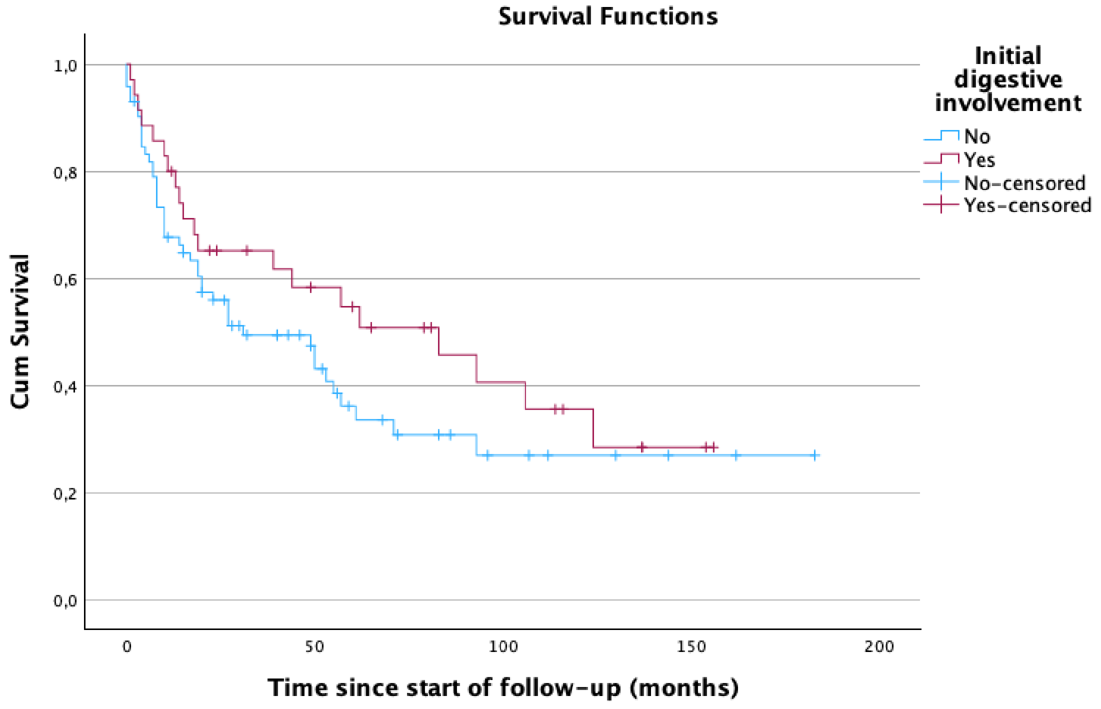

Initial cardiac symptoms

Means and medians for survival

Initial cardiac symptoms	Mean[a]			Median		
	Estimate	Std. Error	(95% CI)	Estimate	Std. Error	(95% CI)
0	65.229	8.850	(47.884, 82.575)	49.000	15.167	(19.273, 78.727)
1	89.075	12.936	(63.721, 114.429)	57.000	—	—
Overall	74.173	7.931	(58.628, 89.719)	50.000	11.725	(27.018, 72.982)

a. Estimation is limited to the largest survival time if it is censored.

Overall Comparisons

	Chi-Square	df	P value
Log Rank (Mantel-Cox)	2.765	1	0.096

Test of equality of survival distributions for the different levels of Initial cardiac symptoms.

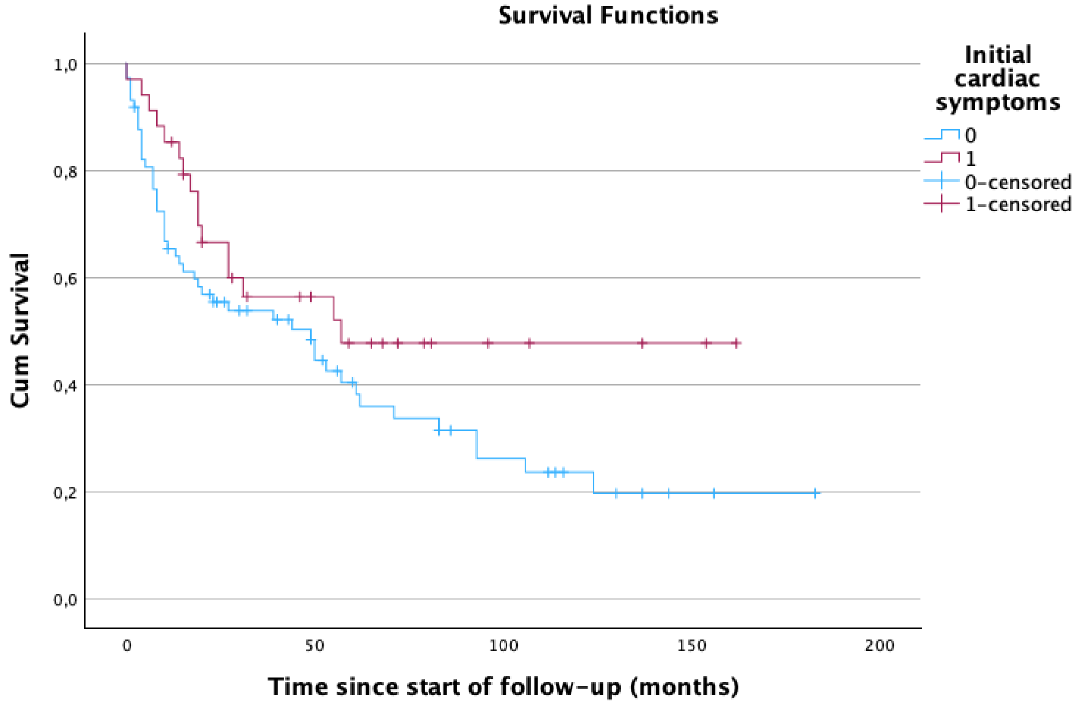

Initial digestive symptoms

Means and medians for survival

Initial digestive symptoms	Mean[a]			Median		
	Estimate	Std. Error	(95% CI)	Estimate	Std. Error	(95% CI)
0	67.838	9.270	(49.669, 86.008)	49.000	13.216	(23.097, 74.903)
1	80.614	12.552	(56.012, 105.215)	83.000	46.605	(0.000, 174.346)
Overall	74.173	7.931	(58.628, 89.719)	50.000	11.725	(27.018, 72.982)

a. Estimation is limited to the largest survival time if it is censored.

Overall Comparisons

	Chi-Square	df	P value
Log Rank (Mantel-Cox)	1.178	1	0.278

Test of equality of survival distributions for the different levels of Initial digestive symptoms.

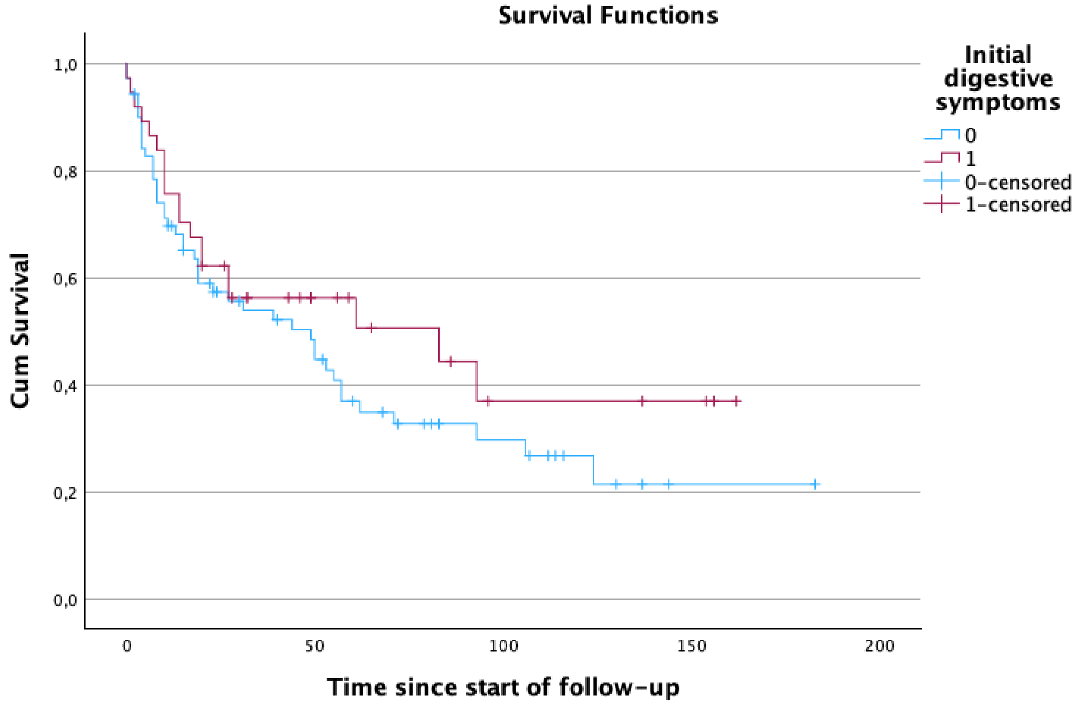

Obesity

Means and medians for survival

	Mean[a]			Median		
Obesity	Estimate	Std. Error	(95% CI)	Estimate	Std. Error	(95% CI)
No	72.324	8.123	(56.403, 88.245)	50.000	12.487	(25.526, 74.474)
Yes	80.278	25.240	(30.807, 129.748)	44.000	24.000	(0.000, 91.040)
Overall	74.173	7.931	(58.628, 89.719)	50.000	11.725	(27.018, 72.982)

a. Estimation is limited to the largest survival time if it is censored.

Overall Comparisons

	Chi-Square	df	P value
Log Rank (Mantel-Cox)	0.614	1	0.433

Test of equality of survival distributions for the different levels of obesity.

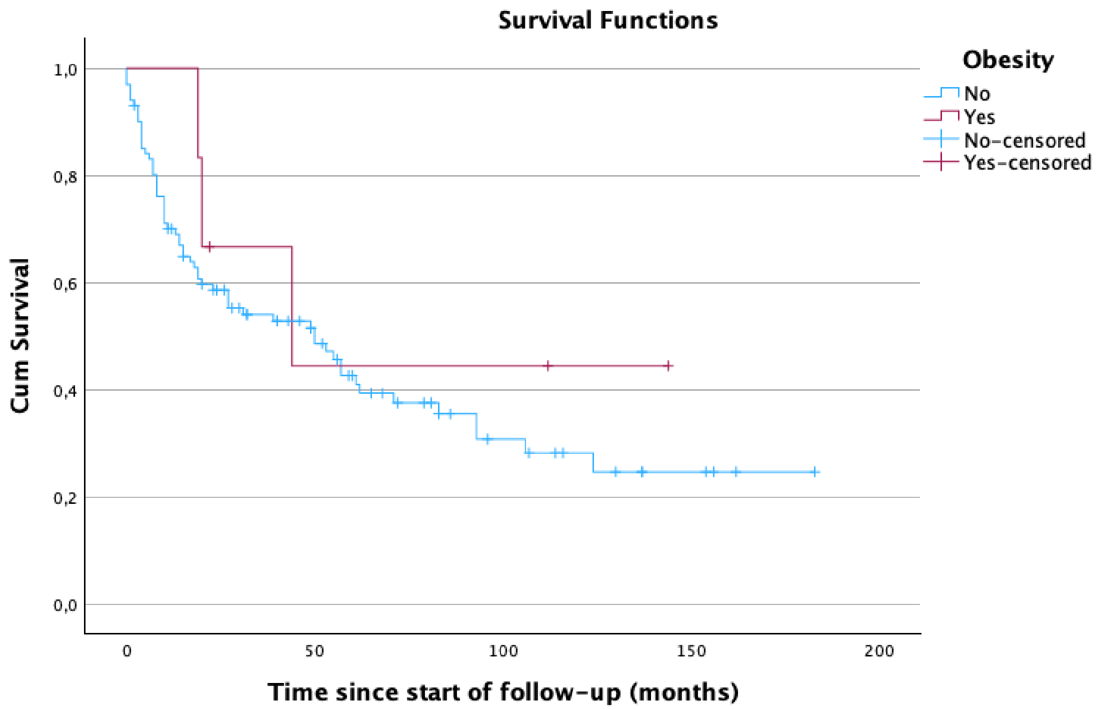

Supplement: Supplementary file 1 [file tropicalmed-10-00161-s001.zip › tropicalmed-3674274-File S1.pdf]
